# Evolution of Perihematomal Edema Mean Hounsfield Unit and Its Association with Clinical Outcome in Intracerebral Hemorrhage: A Post Hoc Analysis of the i-DEF Trial

**DOI:** 10.1007/s12028-025-02337-7

**Published:** 2025-08-11

**Authors:** Alexandros A. Polymeris, Vasileios-Arsenios Lioutas, Diego Incontri, Salil Soman, Magdy H. Selim

**Affiliations:** 1 Stroke Division, Department of Neurology, Beth Israel Deaconess Medical Center, Harvard Medical School, Boston, MA USA; 2 Department of Neurology and Stroke Center, University Hospital Basel and University of Basel, Basel, Switzerland; 3 Department of Radiology, Beth Israel Deaconess Medical Center, Harvard Medical School, Boston, MA USA

**Keywords:** Perihematomal edema, Mean hounsfield unit, Hypodensity, Intracerebral hemorrhage, Outcome

## Abstract

**Background:**

Lower mean Hounsfield unit (mHU) values, indicating greater computed tomography (CT) hypodensity of perihematomal edema (PHE), have been proposed as a novel quantitative imaging marker in intracerebral hemorrhage (ICH). We explored its evolution and prognostic importance in a post hoc analysis of the Intracerebral Hemorrhage-Deferoxamine trial (NCT02175225).

**Methods:**

We included participants with primary supratentorial ICH who had available CT scans at baseline and follow-up after 72–96 h and 90-days and/or 180-days outcome data. The primary exposure variable was the mHU of PHE measured on the follow-up CT scan. We investigated (1) its change from baseline and (2) its association with unfavorable outcome (modified Rankin Scale score 3–6) in adjusted mixed-effects models, accounting for between-center and between-participant variability.

**Results:**

Among 273 of 293 Intracerebral Hemorrhage-Deferoxamine trial participants eligible for analysis (median age 61 years, 39% female), the median (interquartile range) mHU of PHE was 30.3 (28.3–32.7) at baseline and 26.9 (24.6–29.2) at follow-up. Τhe mHU of PHE decreased from baseline to follow-up scan by an average of 3.6 (95% confidence interval [CI] 3.2–4.0, *p* < 0.001). There was no association between the mHU of follow-up PHE with unfavorable outcome at 90 days (*n* = 273; odds ratio 1.05, 95% CI 0.95–1.17, *p* = 0.32), or at 180 days (*n* = 261; odds ratio 1.01, 95% CI 0.92–1.11, *p* = 0.81).

**Conclusions:**

Perihematomal edema after ICH tends to grow more hypodense on CT by day 3–4 compared with baseline. The degree of PHE hypodensity was not associated with long-term clinical outcomes in the setting of a multicenter randomized trial, challenging its utility as a radiological marker in ICH research.

**Clinical Trial Registration:**

ClinicalTrials.gov: NCT02175225.

**Supplementary Information:**

The online version contains supplementary material available at 10.1007/s12028-025-02337-7.

## Introduction

Secondary brain injury from toxicity of hemoglobin-degradation products and inflammatory processes is a potential treatment target in intracerebral hemorrhage (ICH), and perihematomal edema (PHE) is considered its radiological surrogate [1, 2]. On computed tomography (CT), PHE appears as a hypodense area surrounding the ICH lesion. It is typically quantified using volume metrics such as absolute PHE volume, relative PHE, or edema extension distance [1, 3]. So far, research on PHE has almost exclusively focused on these volume metrics. Their natural course in the first days to weeks after ICH is well understood [4–6], and multiple investigations of their association with clinical outcome have been conducted [3]. However, the available evidence regarding the prognostic importance of these PHE volume metrics is inconsistent [3], which limits their usefulness as a radiological surrogate measure of treatment efficacy [1]. To that end, better radiological surrogates of secondary brain injury are needed.

Beyond these measures of PHE volume or extent, hardly any data exist about other attributes of the perihematomal lesion [7]. The degree of PHE hypodensity on CT, which corresponds to the water content in the perihematomal lesion, might reflect the intensity of the secondary injury processes driving PHE formation. As such, the radiodensity (i.e., Hounsfield unit [HU]) values of PHE may capture a different dimension in its pathophysiology, which goes beyond its mere volume or extent, but data about the natural course of the degree of PHE hypodensity are scarce, as are data about its prognostic value. Huan et al. [8] previously investigated the prognostic importance of HU values of PHE; in a retrospective, single-center, observational study, they reported that the mean HU (mHU) value of PHE at 72 h after ICH onset was inversely associated with poor clinical outcome, and appeared to be a superior outcome predictor compared to PHE volume metrics. To our knowledge, these findings have not been validated in other cohorts. Besides preliminary data that radiodensity metrics of PHE might have some value in discriminating neoplastic from nonneoplastic ICH [9, 10], literature on the prognostic value of mHU of perifocal edema surrounding ICH or other focal brain pathologies is altogether lacking.

Therefore, here we (1) investigated the evolution of the mHU of PHE within 72–96 h after ICH and (2) aimed to validate the association of mHU with long-term clinical outcome using high-quality, prospectively collected data from the Intracerebral Hemorrhage-Deferoxamine (i-DEF) trial [11].

## Methods

### Study Design and Participants

The Intracerebral Hemorrhage-Deferoxamine trial (NCT02175225) was a successfully completed, futility-design, randomized, placebo-controlled, phase 2 clinical trial conducted between 2014 and 2017 across 40 centers in the United States and Canada. Its methodology, including details about data collection and neuroimaging analysis, is described elsewhere [11, 12]. In brief, i-DEF randomly assigned participants aged 18–80 years with primary supratentorial ICH to receive daily infusions of deferoxamine mesylate or placebo for 3 consecutive days, starting within 24 h of ICH onset. Patients with planned hematoma evacuation, infratentorial, or secondary ICH were excluded from participation. Noncontrast head CT was obtained at screening (baseline scan) and at 24 (± 6) hours of completion of the last infusion (i.e., within 72–96 h from ICH onset; follow-up scan), using the same standard imaging protocol and scanner at a given center. The guidelines for CT imaging standardization were outlined in a trial-specific imaging protocol, which recommended scanning from the level of the foramen magnum up to the high vertex region in a plane 30° to the infraorbital meatal line, with a 5-mm slice thickness, 5-mm slice spacing, 512 × 512 matrix size, 20–25-cm field of view, a window width of 80–100 HU (supratentorial) or 100–120 HU (posterior fossa) and level of 35–45 HU, and a scanning time of at least 300 mAs. CT scans were sent to a core imaging laboratory (Beth Israel Deaconess Medical Center, Boston, MA). All original imaging evaluations were done by experienced anonymized raters using an imaging analysis software (Analyze version 11.0; AnalyzeDirect, Overland Park, KS). Volumetric assessments of intracerebral hematoma and PHE were done on CT scans using a validated, semiautomated segmentation approach, described in detail previously [11, 12]. In short, areas of hematoma and PHE were automatically delineated on each slice using an edge detection tool guided by rater-adjustable density thresholds (i.e., using the preestablished ranges of 44–100 HU for hematoma and ≤ 35 HU for PHE as reference values [13], with manual adjustment of these thresholds permitted to optimize lesion delineation [14]). This was followed by further manual adjustment of the lesion boundaries in three-dimensional view [14]. The total volumes of hematoma and PHE were then calculated by the software by summing the segmented regions across all slices. Clinical follow-up evaluations included functional status on the modified Rankin Scale (mRS) up to 180 days by certified masked assessors.

In this exploratory post hoc analysis focusing on the mHU of PHE, we included all i-DEF participants who had complete neuroimaging evaluation (both baseline and follow-up CT scans). We excluded those with emergent surgical treatment (i.e., craniectomy/hematoma evacuation), as this may impact PHE, and those without clinical outcome data.

### Exposures and Outcomes

**Fig. 1:**
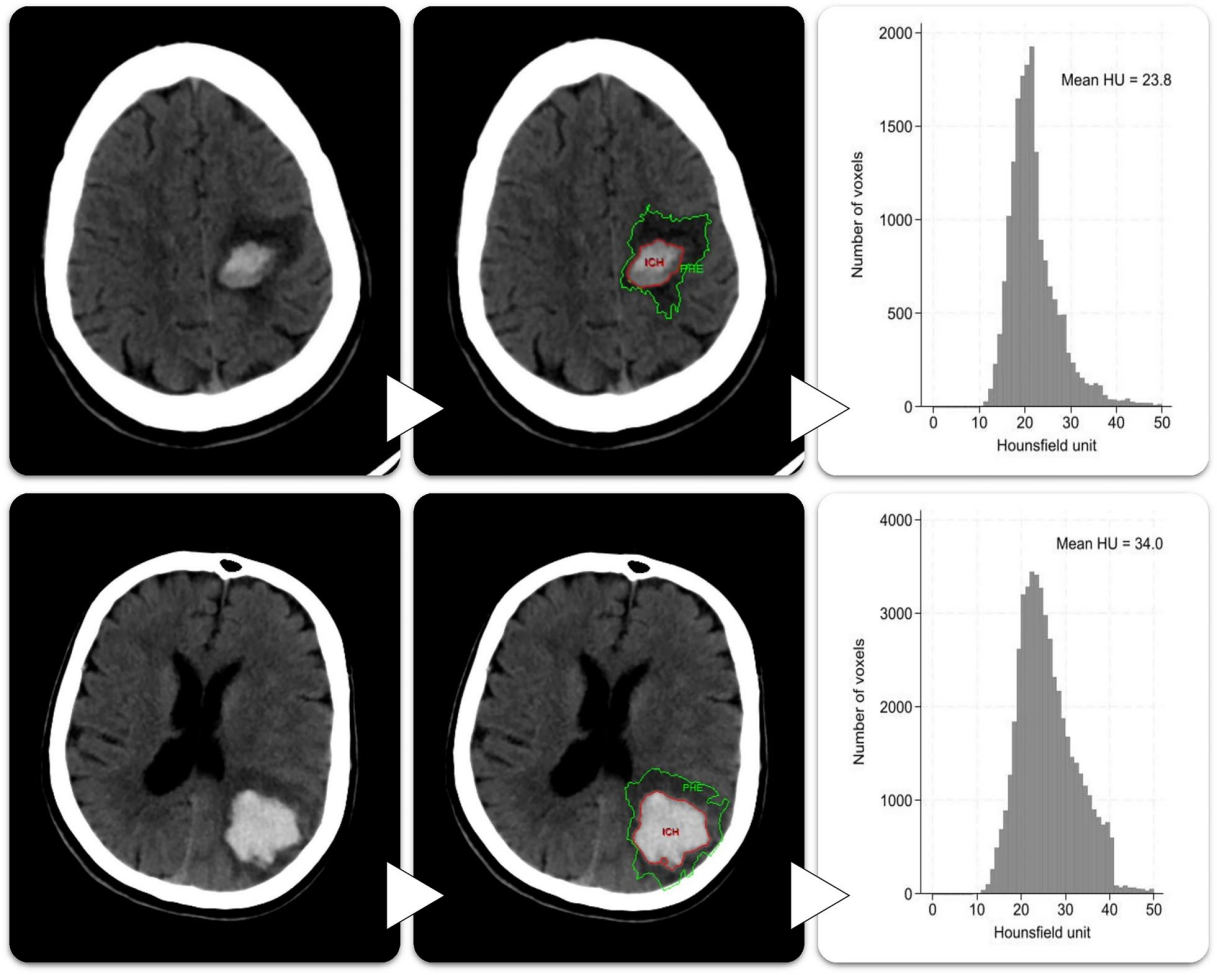
Calculation of the mean HU of PHE in two illustrative cases with low and high values from the same trial center using Analyze 12.0 software (window width 80 HU and level 50 HU). HU, Hounsfield unit, ICH, intracerebral hemorrhage, PHE, perihematomal edema

The primary exposure variable was the mHU of PHE on the follow-up scan (mHU_FU_). For the purposes of this study, we calculated the mHU de novo using the same imaging analysis software (Analyze version 12.0) as for the trial’s original volumetric assessments [11, 12]. After loading the previously segmented volumetric data onto the participants’ CT image data sets, the mHU of PHE was output by the software as a summary measure of the density of all voxels included within the respective PHE volume, as illustrated in Fig. 1, in accordance with previous research [15, 16]. We also calculated the mHU of PHE on the baseline scan (mHU_BL_)—a variable crucial to investigate the evolution of PHE and to account for interindividual variability and other biases—in the same fashion. From those, we also calculated the difference of the mHU of PHE between the baseline and follow-up scan (mHU_d_) for each participant, according to the formula mHU_d_ = mHU_FU_ − mHU_BL_.

The main outcome was unfavorable functional outcome at 90 days, defined as an mRS score 3–6, in keeping with the main trial [11]. Secondary outcome was unfavorable functional outcome at 180 days.

### Statistical Analyses

We grouped participants according to unfavorable 90-day outcome and presented their characteristics using descriptive statistics (frequencies and percentages, medians with interquartile ranges [IQRs], and/or means with standard deviations [SDs] for categorical and continuous data, respectively). We compared categorical and continuous variables using *χ*^2^ or Fisher’s exact tests and Mann–Whitney *U*-test or *t*-tests as appropriate, respectively.

### Evolution of the mHU of PHE

To explore the evolution of the mHU of PHE from baseline to follow-up scan, we used (1) Pearson’s correlation coefficient to test the correlation between mHU_BL_ and mHU_FU_; (2) a paired t-test to compare mHU_BL_ versus mHU_FU_; and (3) a linear mixed model with mHU of PHE as a repeatedly measured continuous outcome (at two time points, baseline and follow-up). The model included center identifier (ID) and nested participant ID as random intercepts and time point as a fixed effect. We adjusted this model for baseline PHE volume, baseline serum glucose, use of hyperosmolar therapy (hypertonic saline and/or mannitol), and actual i-DEF treatment received (deferoxamine or placebo). There were no missing values in any of these covariates. This approach evaluates, again, the change of mHU from baseline to follow-up while accounting not only for the intraindividual correlation of repeated measures but also for between-center (and between-participant) differences, as well as for pathophysiological and treatment-related factors potentially influencing PHE. We did not include ICH volume as a covariate because this is known to be highly correlated to PHE volume. In sensitivity analyses, we fitted the model again by alternatively adjusting for baseline volume of ICH instead of PHE and additionally adjusting for baseline National Institutes of Health Stroke Scale or Glasgow Coma Scale (GCS) score. In the absence of information about specific CT scanner types and the actual imaging parameters used in trial participants, we used trial centers as a proxy to estimate measurement variability, i.e., systematic noise, potentially related to site-specific image acquisition and reconstruction parameters, and report its intraclass correlation coefficient as a measure of the proportion of the overall variance in mHU measurements attributable to between-center variability.

### Correlation of mHU of PHE with ICH and PHE Volume

We explored the correlation of the mHU of PHE with the volume of ICH and PHE both at baseline and at follow-up using Spearman’s rank correlation coefficient.

### Association of mHU of PHE with Unfavorable Outcome

For this, we fitted a generalized linear mixed-effects model with logit link function and the main outcome (unfavorable 90-day outcome) as dependent variable. We fitted three iterations of this model to account for baseline interindividual differences:

1. We used mHU_FU_ as independent variable and included adjustment for mHU_BL_. To address potential imprecision or instability of the model-based estimates related to collinearity between mHU_FU_ and mHU_BL_, we used the following additional approaches:
2. We used mHU_d_ as an independent variable, without adjustment for mHU_BL_, and
3. We regressed mHU_FU_ on mHU_BL_ in a separate linear model and investigated the association of the resulting mHU_FU_ residuals with unfavorable functional outcome in a third iteration of our model that did not include mHU_BL_ as an adjusting covariate. The estimate of this model for the effect of the mHU_FU_ residuals on unfavorable functional outcome can be interpreted as an estimate for the effect of mHU_FU_ on outcome while controlling for mHU_BL_. This approach has been used in previous PHE research to address collinearity issues [17].

To account for center-level sources of measurement noise and outcome heterogeneity, we included center ID as random intercept in all models. All models were additionally adjusted for established independent outcome predictors including age, GCS score at presentation, and ICH volume as continuous variables, ICH location (lobar, thalamic, or deep nonthalamic) and intraventricular hemorrhage extension (present vs. absent) as categorical variables [18], as well as i-DEF treatment received.

In sensitivity analyses, we further adjusted for sex and tested for potential interaction between mHU of PHE and ICH volume in all three model iterations. Moreover, we conducted an additional sensitivity analysis in which all three model iterations described earlier were repeated using the mHU of PHE normalized to the corresponding mHU of the hematoma (i.e., by dividing the mHU of PHE by the mHU of ICH from the same scan), as a further attempt to control for measurement variability. The mHU of ICH required for this was calculated in the same way as described earlier for the mHU of PHE. To improve interpretability of the estimates, we applied a simple linear scaling transformation (multiplication by 100) to normalized mHU values in the models.

Finally, we repeated these analyses using 180-day outcome. Considering the low missingness rate of 180-day mRS data in i-DEF, these analyses were done on a complete case basis.

For all analyses, we report model‐based estimates as β coefficients or odds ratios (ORs) along with 95% confidence intervals (CIs) and two-sided *p* values. We applied no multiplicity correction, given the exploratory nature of the analysis. Analyses were done using STATA version 19.5 (StataCorp LLC, College Station, TX). We report this study in accordance with the Strengthening the Reporting of Observational Studies in Epidemiology statement.

## Results

Of 293 i-DEF participants, 1 lacked baseline imaging assessment, 8 underwent emergent surgical treatment, 6 lacked follow-up imaging assessment, and 5 were missing 90-day outcome data, leaving 273 (93%) participants available for analysis (median age 61 years, 39% female). All clinical, laboratory, and radiological data at baseline and follow-up are given in Table 1.

**Table 1:** Participant characteristics

	All (*N* = 273)	Favorable 90-d outcome (*n* = 94)	Unfavorable 90-d outcome (*n* = 179)	*p* value
Age, median (IQR) (yr)	61 (52–70)	58 (50–68)	61 (54–71)	0.10
Female sex, *n* (%)	105 (38.5)	27 (28.7)	78 (43.6)	0.02
Medical history, *n* (%)				
Hypertension	225 (82.4)	75 (79.8)	150 (83.8)	0.41
Diabetes mellitus	74 (27.1)	28 (29.8)	46 (25.7)	0.47
Cardiac disease	27 (9.9)	10 (10.6)	17 (9.5)	0.76
Previous ischemic stroke	25 (9.2)	11 (11.7)	14 (7.8)	0.29
Previous ICH	10 (3.7)	2 (2.1)	8 (4.5)	0.33
Clinical, laboratory, and radiological data at baseline				
GCS score, median (IQR)	14 (12–15)	15 (13–15)	14 (11–15)	< 0.001
NIHSS score, median (IQR)	13 (8–18)	8 (7–12)	15 (11–19)	< 0.001
Serum glucose, median (IQR) (mg/dL)	137 (116–162)	131.5 (113–164)	139 (117–161)	0.48
ICH onset to baseline CT scan, median (IQR) (hr)	3.3 (1.3–7.1)	3.3 (1.5–6.7)	3.3 (1.3–7.1)	0.97
ICH location, *n* (%)				0.07
Lobar	48 (17.6)	20 (21.3)	28 (15.6)	
Thalamic	104 (38.1)	27 (28.7)	77 (43.0)	
Deep nonthalamic	121 (44.3)	47 (50.0)	74 (41.3)	
ICH volume, median (IQR) (mL)	12.2 (6.1–23.5)	8.8 (5.7–14.3)	15.5 (6.9–27.9)	< 0.001
mHU of ICH, median (IQR)	58.9 (56.0–62.0)	58.1 (55.1–61.3)	60.2 (56.7–62.6)	0.010
Intraventricular hemorrhage extension, *n* (%)	113 (41.4)	27 (28.7)	86 (48.0)	0.002
PHE volume, median (IQR) (mL)	14.6 (8.3–23.4)	11.5 (7.1–17.3)	15.9 (9.0–29.1)	< 0.001
mHU of PHE, median (IQR)	30.3 (28.3–32.7)	30.1 (27.8–32.6)	30.4 (28.7–32.8)	0.23
mHU of PHE normalized to mHU of ICH, median (IQR)	0.51 (0.48–0.55)	0.51 (0.49–0.54)	0.51 (0.48–0.55)	0.39
Treatment, *n* (%)				
i-DEF treatment received				0.91
Placebo	132 (48.4)	45 (47.9)	87 (48.6)	
Deferoxamine	141 (51.6)	49 (52.1)	92 (51.4)	
Use of hyperosmolar therapy	36 (13.2)	4 (4.3)	32 (17.9)	0.002
Radiological data at follow-up				
ICH onset to follow-up CT scan, median (IQR) (hr)	88.7 (81.1–94.8)	87.9 (80.5–93.8)	89.0 (81.2–95.1)	0.45
Baseline to follow-up CT scan, median (IQR) (hr)	82.4 (77.0–90.1)	82.4 (76.6–88.4)	82.4 (77.2–90.8)	0.52
ICH volume, median (IQR) (mL)	11.6 (6.2–23.6)	8.6 (5.3–15.0)	15.7 (7.2–27.2)	< 0.001
mHU of ICH, median (IQR)	58.4 (54.7–61.4)	56.5 (54.0–60.4)	58.8 (55.7–61.9)	0.005
PHE volume, median (IQR) (mL)	22.8 (14.9–8.4)	20.0 (13.0–31.8)	25.8 (15.4–47.1)	< 0.001
mHU of PHE, median (IQR)	26.9 (24.6–29.2)	26.4 (24.5–28.8)	27.2 (24.6–29.4)	0.15
mHU of PHE normalized to mHU of ICH, median (IQR)	0.46 (0.43–0.49)	0.46 (0.44–0.49)	0.46 (0.43–0.49)	0.77
mHU of PHE interscan difference, median (IQR)	− 3.5 (− 5.4 to − 2.1)	− 3.3 (− 5.1 to − 2.2)	− 3.6 (− 5.5 to − 2.0)	0.93
mHU of PHE normalized to mHU of ICH interscan difference, median (IQR)	− 0.05 (− 0.08 to − 0.02)	− 0.05 (− 0.08 to − 0.03)	− 0.05 (− 0.08 to − 0.02)	0.93

*CT* computed tomography; *ICH* intracerebral hemorrhage; *i-DEF* Intracerebral Hemorrhage-Deferoxamine; *IQR* interquartile range; *GCS* Glasgow Coma Scale; *mHU* mean Hounsfield unit; *NIHSS* National Institutes of Health Stroke Scale; *PHE* perihematomal edema

### Evolution of the mHU of PHE

**Fig. 2:**
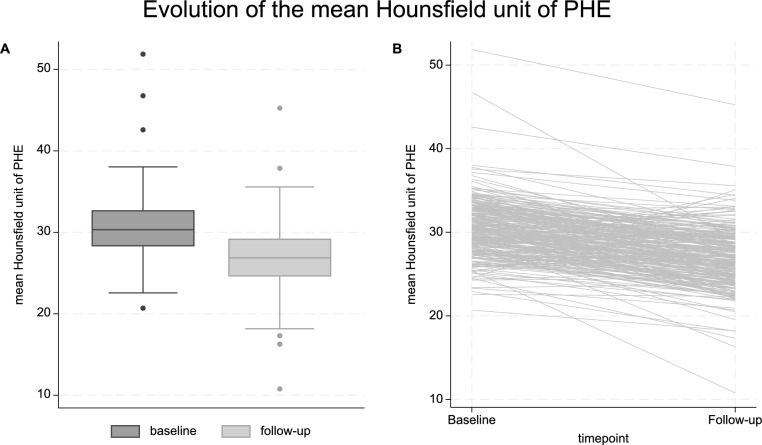
Evolution of the mean Hounsfield unit (mHU) of PHE from baseline to follow-up scan (panel **A** shows the boxplots of mHU at both timepoints; panel **B** shows a line plot of the individual changes in mHU from baseline to follow-up for each participant). PHE, perihematomal edema

Baseline CT was done at a median (IQR) of 3.3 (1.3–7.1) hours after ICH onset, and follow-up CT at 88.8 (81.1–94.8) hours. The median (IQR) for mHU_BL_ was 30.3 (28.3–32.7) and for mHU_FU_ was 26.9 (24.6–29.2). At both time points, mHU was nearly normally distributed (Fig. 2A), with a mean (± SD) mHU_BL_ of 30.5 (± 3.5) and mHU_FU_ of 26.9 (± 3.6). This amounted to an mHU_d_ with a median (IQR) of − 3.5 (− 5.4 to − 2.1) and mean (± SD) of − 3.6 (± 3.5).

There was moderate correlation between mHU_BL_ and mHU_FU_ (Pearson’s *ρ* = 0.52, *p* < 0.001). A paired *t*-test revealed a significant decrease of mHU_FU_ compared with mHU_BL_ (*p* < 0.001). In the adjusted linear mixed model including both mHU_BL_ and mHU_FU_ as repeatedly measured outcome, as well as trial center and participant as random effects, the mHU of PHE decreased with time, being on average about 3.5 units lower at follow-up compared with baseline (β = − 3.6, 95% CI − 4.0 to − 3.2, *p* < 0.001; Fig. 2B). Results were unchanged in sensitivity analyses alternatively adjusting the model for baseline volume of ICH instead of PHE, and also after additionally adjusting the model for baseline National Institutes of Health Stroke Scale or GCS score (Supplementary Table 1). The intraclass correlation coefficient for trial center was 21.8%, indicating that approximately 1/5 of the total variance in mHU measurements was attributable to between-center variability, potentially reflecting measurement noise.

### Correlation of mHU of PHE with ICH and PHE Volume

There was no correlation of the mHU of PHE with the volume of ICH (Spearman’s *ρ* = 0.03, *p* = 0.63) or the volume of PHE (Spearman’s *ρ* = − 0.08, *p* = 0.17) at baseline. At follow-up, there was no clear correlation between the mHU of PHE and ICH volume or PHE volume (*ρ* = 0.12, *p* = 0.05 and *ρ* = 0.03, *p* = 0.63, respectively).

### Association of mHU of PHE with Long-Term Clinical Outcome

**Fig. 3:**
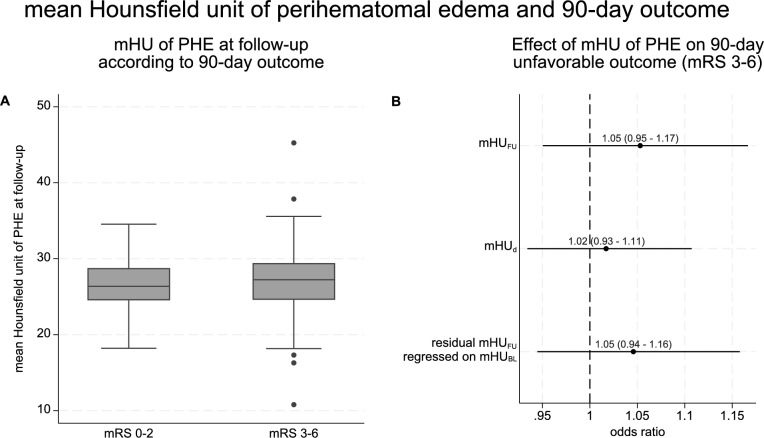
Association of the mHU of PHE with 90-day unfavorable clinical outcome (panel **A** shows the distribution of mHU at follow-up according to unfavorable clinical outcome; panel **B** shows model-based estimates for the association of mHU with 90-day outcome). mHU, mean Hounsfield unit, mHU_BL_, mean Hounsfield unit of perihematomal edema on the baseline scan, mHU_d_, difference of the mean Hounsfield unit of perihematomal edema between the baseline and follow-up scan, mHU_FU_, mean Hounsfield unit of perihematomal edema on the follow-up scan, PHE, perihematomal edema

Of 273 participants, 94 (34%) had a favorable outcome (mRS 0–2) and 179 (66%) had an unfavorable outcome (mRS 3–6) at 90 days. Figure 3A shows the distribution of mHU_FU_ according to outcome. In univariate analysis, mHU_FU_ did not differ between participants with favorable versus unfavorable 90-day outcome (median [IQR] 26.4 [24.5–28.8] vs. 27.2 [24.6–29.4], Mann–Whitney *U*-test *p* = 0.15; mean [± SD] 26.5 [± 3.1] vs. 27.1 [± 3.8]; *t*-test *p* = 0.24). In the multivariable generalized linear mixed model, mHU_FU_ was not associated with 90-day unfavorable outcome after accounting for trial center as random effect and adjusting for mHU_BL_, randomized treatment and established outcome predictors (OR 1.05, 95% CI 0.95–1.17, *p* = 0.32). The additional model iterations using mHU_d_ and residual mHU_FU_ regressed on mHU_BL_ yielded similar results, without evidence of association between mHU and clinical outcome (Fig. 3B). Sensitivity analyses across all three model iterations revealed similar findings after additional adjustment for sex (Supplementary Table 2) and no evidence for modification of the association between mHU of PHE and 90-day outcome by ICH volume (p_interaction_ = 0.12, 0.96, and 0.41, respectively). Sensitivity analyses after normalization of the mHU of PHE to the mHU of ICH revealed, again, no signal for an association with 90-day outcome (Supplementary Fig. 1).

Repeated analyses using 180-day outcomes (available in 261/273 participants [96%]) showed similar findings in univariate analysis (mHU_FU_ in participants with favorable vs. unfavorable 180-day outcome: median [IQR] 26.7 [24.6–28.8] vs. 27.0 [24.8–29.5], Mann–Whitney *U*-test *p* = 0.35; mean [SD] 26.7 [± 3.0] vs. 27.0 [± 4.0]; *t*-test *p* = 0.40) and in the multivariable models (OR 1.01, 95% CI 0.92–1.11, *p* = 0.81 for mHU_FU_; Supplementary Fig. 2).

## Discussion

This exploratory post hoc analysis of the prospective multicenter i-DEF trial focusing on the mHU of PHE as a potential new radiological marker in ICH revealed the following key findings: the mHU of PHE decreased significantly over the first 3–4 days after ICH, and the mHU of PHE at follow-up was not associated with long-term functional outcome, challenging its usefulness as a radiological surrogate.

Our finding that the mHU of PHE significantly decreases with time is biologically plausible given previous knowledge about PHE pathophysiology and evolution [1]. The prevailing paradigm holds that the perihematomal lesion expands rapidly in volume within the first few days after ICH, owing to increasing vasogenic and cytotoxic edema [1, 2, 4, 5]. It is not surprising that, as the processes driving PHE intensify within this timeframe, not only the volume but also the degree of hypodensity on CT increases, reflecting the increasing water content of the perihematomal lesion. This finding elucidates a previously unexplored dimension in the radiological evolution of PHE, expanding on existing data regarding its natural history [4–6].

Although this finding supports the biological relevance of the mHU of PHE, our analyses exploring its association with clinical outcome found no signals for such association with 90-day or 180-day clinical outcome. This was true in univariate analyses and persisted after controlling for potential confounding through known outcome predictors and after using several approaches to account for between-center differences (by including center as random effect) and for between-participant differences (by incorporating the mHU of baseline PHE in our analyses), and even after standardizing the mHU of PHE to the mHU of the hematoma as an effort to account for measurement variability.

This contradicts the findings of Huan et al. [8], who, in a smaller single-center study (< 200 patients), reported an association between follow-up PHE mHU and clinical outcome, suggesting it might even be superior to PHE volume metrics as a predictor. In the i-DEF data set, volume metrics of PHE have previously been shown to have only a weak association with outcome [12]. Unlike the study by Huan et al. [8]*,* which was single-center, observational, and retrospective, i-DEF used standardized and centralized assessments, prospective design, and rigorous predefined times for follow-up scans. These differences may account for the discrepant results.

An alternative explanation for the lack of association of the mHU of PHE with clinical outcome in our study may lie in the large number of sites enrolling participants in i-DEF. CT scanners of different manufacturers and models with varying reconstruction algorithms as well as differences in local calibration and maintenance practices may have introduced inconsistencies in mHU measurements and noise to our analyses [19]. Indeed, we estimated that a substantial proportion (~ 20%) of the overall variance in mHU measurements was attributable to between-center differences and may thus reflect systematic noise from imaging disparities across sites. Despite trial-specific CT imaging standardization recommendations and several different statistical approaches to account for measurement noise, this may have still obscured any association of the mHU of PHE with outcome in our data, in contrast to Huan et al. [8], who used a single scanner in their study. It must be noted, however, that previous research not on PHE but on hematoma HU in various types of intracranial hemorrhage was done using data across multiple CT scanners [15, 16, 20, 21], including research in multicenter settings [22]. Despite possible noise arising from potential measurement inconsistencies, these studies were able to demonstrate that the hematoma’s HU values bore clinical significance [15, 16, 20–23], in contrast to PHE’s mHU in our investigation.

Regardless of the reasons for the lack of association of the mHU of PHE with clinical outcome in our study, our findings may have implications for future research in ICH: our data currently provide no support for the use of the mHU of PHE as a surrogate measure of treatment efficacy in the setting of multicenter trials and challenge its overall utility as a radiological surrogate. Future research on the mHU of PHE in multicenter settings will need to minimize imaging disparities across centers, so as to provide an unbiased and definitive appraisal of the prognostic value of the mHU of PHE in the absence measurement noise. In the meantime, other features of PHE beyond volume, such as its shape or homogeneity, might be more robust to variability in multicenter settings and prove more useful [7].

Strengths of our study include the high quality of data collected prospectively in the setting of a randomized trial with rigorous and standardized methodology and central imaging assessment. However, our study is limited in that (1) it lacked information on the types of scanners used at each trial center and each center’s adherence to imaging standardization recommendations. Despite best efforts to account for between-center and between-participant variability, we can ultimately not exclude that our findings are influenced by disparities in image acquisition and reconstruction; (2) its exploratory post hoc nature positions our findings as hypothesis-generating rather than definitive; (3) its patient population was selected using specific eligibility criteria in the setting of a randomized trial excluding patients with infratentorial, surgically treated, and secondary ICH. Our cohort might thus be less reflective of the broader population of patients with ICH, to which our finding may not generalize; (4) our analyses of clinical outcome accounted only for the best established independent outcome predictors, but did not include adjustment for comorbidities or other potential outcome modifiers.

## Conclusions

Perihematomal edema after ICH tends to grow more hypodense on CT by day 3–4 compared with baseline. In a post hoc analysis of the i-DEF trial, the degree of its hypodensity was not associated with long-term clinical outcome, challenging the use of the mHU of PHE as a radiological outcome in ICH research.

## Conflict of interest

Dr. Lioutas receives support from the NIH, NINDS, and National Institute on Aging (U01NS102289; UF1NS120871). Dr. Selim receives grant funding/support from the NIH, NINDS and National Institute on Aging (U01NS102289; UF1NS120871; UG3NS128397); serves as an advisory board member/ consultant for MedRhythms, Inc, AegisCN, LLC, and Alnylam Pharmaceuticals; receives royalties from UpToDate, Inc. and Oxford University Press; and holds equity at NeuGel Therapeutics, Inc. The remaining authors report no conflicts of interest.

## Ethical Approval

All Intracerebral Hemorrhage-Deferoxamine participants (or a legally authorized representative) provided written informed consent and the trial was approved by the local ethics committee and institutional review board at each participating center.

## Supplementary Information

Below is the link to the electronic supplementary material.

Supplementary file1 (DOCX 59 KB)

Supplementary file2 (PDF 182 KB)
