# Supplementary material for: Evolution of Perihematomal Edema Mean Hounsfield Unit and Its Association with Clinical Outcome in Intracerebral Hemorrhage: A Post Hoc Analysis of the i-DEF Trial
Source: Neurocrit Care. 2025 Aug 11;43(3):824–33. doi: 10.1007/s12028-025-02337-7 (PMC12647195; doi:10.1007/s12028-025-02337-7)
Supplement: Supplementary file 1 — Supplementary file1 (DOCX 59 KB) [file 12028_2025_2337_MOESM1_ESM.docx]

**Supplementary Material**

**Evolution of perihematomal edema mean Hounsfield unit and its association with clinical outcome in intracerebral hemorrhage: a post hoc analysis of the i-DEF trial**

Polymeris AA, Lioutas VA, Incontri D, Soman S, Selim MH, on behalf of the i-DEF Investigators

| **Supplementary Table 1.** Multivariable linear mixed model with mHU of PHE as a repeatedly measured continuous outcome (at 2 timepoints, baseline and follow-up). Center ID and nested participant ID were included as random intercepts. | | |
| --- | --- | --- |
| **Model/variable** | **β coefficient (95% CI)** | **p-value** |
| **Main model** |  |  |
| Follow-up timepoint (ref: baseline) | -3.56 (-3.97 to -3.15) | <0.001 |
| baseline PHE volume (per 10 mL increase) | 0.35 (0.16 to 0.54) | <0.001 |
| baseline serum glucose (per 10 mg/dL increase) | 0.05 (-0.01 to 0.11) | 0.097 |
| received hyperosmolar therapy | -1.24 (-2.29 to -0.19) | 0.020 |
| received deferoxamine treatment (ref: placebo) | 0.22 (-0.43 to 0.88) | 0.503 |
| **Sensitivity analysis** (alternatively adjusting for baseline volume of ICH instead of PHE) |  |  |
| Follow-up timepoint (ref: baseline) | -3.56 (-3.97 to -3.15) | <0.001 |
| baseline ICH volume (per 10 mL increase) | 0.45 (0.27 to 0.64) | <0.001 |
| baseline serum glucose (per 10 mg/dL increase) | 0.05 (-0.01 to 0.11) | 0.081 |
| received hyperosmolar therapy | -1.47 (-2.51 to -0.43) | 0.005 |
| received deferoxamine treatment (ref: placebo) | 0.27 (-0.38 to 0.91) | 0.416 |
| **Sensitivity analysis** (additionally adjusting for baseline NIHSS score) |  |  |
| Follow-up timepoint (ref: baseline) | -3.56 (-3.97 to -3.15) | <0.001 |
| baseline PHE volume (per 10 mL increase) | 0.39 (0.19 to 0.59) | <0.001 |
| baseline serum glucose (per 10 mg/dL increase) | 0.06 (-0.00 to 0.11) | 0.057 |
| received hyperosmolar therapy | -1.02 (-2.10 to 0.06) | 0.064 |
| received deferoxamine treatment (ref: placebo) | 0.18 (-0.47 to 0.84) | 0.582 |
| baseline NIHSS score (per 5 points increase) | -0.24 (-0.55 to .07) | 0.135 |
| **Sensitivity analysis** (additionally adjusting for baseline GCS score) |  |  |
| Follow-up timepoint (ref: baseline) | -3.56 (-3.97 to -3.15) | <0.001 |
| baseline PHE volume (per 10 mL increase) | 0.38 (0.18 to 0.58) | <0.001 |
| baseline serum glucose (per 10 mg/dL increase) | 0.05 (-0.00 to 0.11) | 0.073 |
| received hyperosmolar therapy | -1.11 (-2.18 to -0.03) | 0.043 |
| received deferoxamine treatment (ref: placebo) | 0.18 (-0.48 to 0.84) | 0.591 |
| baseline GCS score (per 1 point increase) | 0.09 (-0.08 to 0.26) | 0.310 |
| The β coefficient for the adjusting covariates can be interpreted as the average change in the mHU of PHE (across both timepoints, baseline and follow-up) per unit change in the covariate.  mHU, mean Hounsfield Unit; PHE, perihematomal edema; ICH, intracerebral hemorrhage; NIHSS, National Institutes of Health Stroke Scale; GCS, Glasgow Coma Scale | | |

| **Supplementary Table 2.** Multivariable generalized linear mixed-effects models with logit link for unfavorable 90-day outcome with additional covariate adjustment for sex. Center ID was included as random intercept. | | |
| --- | --- | --- |
| **Model iteration/variable** | **Odds ratio (95% CI)** | **p-value** |
| **Iteration (i)** |  |  |
| mHU_FU_ (per 1 HU increase) | 1.07 (0.97 to 1.19) | 0.186 |
| mHU_BL_ (per 1 HU increase) | 1.01 (0.91 to 1.12) | 0.893 |
| age (per 1 year increase) | 1.03 (1.00 to 1.06) | 0.032 |
| presenting GCS score (per 1 point increase) | 0.82 (0.69 to 0.99) | 0.034 |
| ICH volume (per 1 mL increase) | 1.08 (1.04 to 1.11) | <0.001 |
| thalamic ICH location (ref: lobar) | 13.0 (4.0 to 42.3) | <0.001 |
| deep non-thalamic ICH location (ref: lobar) | 4.37 (1.50 to 12.74) | 0.007 |
| IVH extension | 1.14 (0.58 to 2.26) | 0.697 |
| received deferoxamine treatment (ref: placebo) | 1.23 (0.69 to 2.19) | 0.493 |
| female sex (ref: male) | 2.27 (1.22 to 4.22) | 0.010 |
| **Iteration (ii)** |  |  |
| mHU_d_ (per 1 HU increase) | 1.03 (0.94 to 1.12) | 0.511 |
| age (per 1 year increase) | 1.03 (1.00 to 1.05) | 0.053 |
| presenting GCS score (per 1 point increase) | 0.81 (0.68 to 0.98) | 0.026 |
| ICH volume (per 1 mL increase) | 1.07 (1.04 to 1.11) | <0.001 |
| thalamic ICH location (ref: lobar) | 11.5 (3.6 to 36.0) | <0.001 |
| deep non-thalamic ICH location (ref: lobar) | 3.60 (1.30 to 9.91) | 0.013 |
| IVH extension | 1.10 (0.56 to 2.15) | 0.792 |
| received deferoxamine treatment (ref: placebo) | 1.24 (0.69 to 2.21) | 0.474 |
| female sex (ref: male) | 2.19 (1.18 to 4.08) | 0.013 |
| **Iteration (iii)** |  |  |
| residual mHU_FU_ regressed on mHU_BL_ | 1.07 (0.96 to 1.18) | 0.236 |
| age (per 1 year increase) | 1.03 (1.00 to 1.05) | 0.045 |
| presenting GCS score (per 1 point increase) | 0.82 (0.68 to 0.98) | 0.029 |
| ICH volume (per 1 mL increase) | 1.07 (1.04 to 1.11) | <0.001 |
| thalamic ICH location (ref: lobar) | 12.3 (3.8 to 39.2) | <0.001 |
| deep non-thalamic ICH location (ref: lobar) | 3.93 (1.40 to 11.07) | 0.010 |
| IVH extension | 1.11 (0.56 to 2.19) | 0.761 |
| received deferoxamine treatment (ref: placebo) | 1.23 (0.69 to 2.20) | 0.481 |
| female sex (ref: male) | 2.25 (1.21 to 4.20) | 0.011 |
| PHE, perihematomal edema; mHU, mean Hounsfield Unit; mHU_FU_, mHU of PHE at follow-up; mHU_BL_, mHU of PHE at baseline; mHU_d_, difference in mHU of PHE between baseline and follow-up; ICH, intracerebral hemorrhage; IVH, intraventricular hemorrhage; GCS, Glasgow Coma Scale | | |

**Supplementary Figure 1.** Association of the mean Hounsfield unit (mHU) of perihematomal edema (PHE) normalized to the mHU of the intracerebral hematoma (ICH) with 90-day unfavorable clinical outcome.

The normalized mHU of PHE was calculated as the ratio mHU of PHE / mHU of ICH.

Panel A shows the distribution of normalized mHU of PHE at follow-up according to unfavorable clinical outcome.

Panel B shows model-based estimates for the association of normalized mHU of PHE, linearly rescaled by multiplication by 100, with 90-day outcome. Thus, odds ratios reflect the change in odds of unfavorable outcome per 1% increase of the normalized mHU of PHE. Here, mHU_BL_ and mHU_FU_ denote mHU on the baseline and follow-up scan, respectively; mHU_d_ denotes the difference in mHU between baseline and follow-up scan.

**Supplementary Figure 2.** Association of the mean Hounsfield unit (mHU) of perihematomal edema (PHE) with 180-day unfavorable clinical outcome (panel A shows the distribution of mHU at follow-up according to unfavorable clinical outcome; panel B shows model-based estimates for the association of mHU with 180-day outcome; mHU_BL_ and mHU_FU_ denote mHU on the baseline and follow-up scan, respectively; mHU_d_ denotes the difference in mHU between baseline and follow-up scan).
